# New replacement name for *Chrysotus infirmus* Wei, Zhang & Zhou, 2014 (Diptera, Dolichopodidae, Diaphorinae)

**DOI:** 10.3897/zookeys.607.9543

**Published:** 2016-07-28

**Authors:** Zheng-Xiang Zhou

**Affiliations:** 1 College of Agriculture, Anshun University, Anshun, Guizhou, P.R. China, 561000

**Keywords:** Diptera, Dolichopodid, Chrysotus, homonym, replacement name

## Abstract

*Chrysotus weii* Zhou, **nom. n.**, the new replacement name is proposed for the species *Chrysotus infirmus* Wei, Zhang & Zhou, 2014 (Diptera: Brachycera: Dolichopodidae: Diaphorinae), which was preoccupied by *Chrysotus infirmus* Parent, 1933.

## Introduction

Wei, Zhang & Zhou (2014) described the dolichopodid species *Chrysotus infirmus* from Guizhou Province, China. However, the scientific name was preoccupied by *Chrysotus infirmus* Parent, 1933. Thus, the scientific name *Chrysotus infirmus* Wei, Zhang & Zhou, 2014 is a junior primary homonym of the species *Chrysotus infirmus* Parent, 1933. According to Article 60.3 and 57.2 of the ICZN, the new replacement name *Chrysotus weii* Zhou, **nom. n.** for *Chrysotus infirmus* Wei, Zhang & Zhou, 2014 is proposed.

## Result

### Chrysotus weii

Taxon classificationAnimaliaDipteraDolichopodidae

Zhou nom. n.

Chrysotus infirmus Wei, Zhang & Zhou, 2014: 55. Preoccupied by Parent 1933:179.

#### Etymology.

Specific epithet is dedicated to Prof . Wei Lianmeng, for his contributions to the systematic work of Chinese Diptera.

#### Distribution.

China (Guizhou).

##### Summary of nomenclatural changes

*Chrysotus weii* Zhou, **nom. n.**= *Chrysotus infirmus* Wei, Zhang & Zhou, 2014 (nec Parent, 1933)

## Supplementary Material

XML Treatment for Chrysotus weii
